# Clopidogrel versus ticagrelor in the treatment of Chinese patients undergoing percutaneous coronary intervention: effects on platelet function assessed by platelet function tests and mean platelet volume

**DOI:** 10.1186/s12959-021-00350-2

**Published:** 2021-12-07

**Authors:** Yang Zhang, Rui Peng, Xiaojuan Li, Gaowa Cheng, Ximing Wang, Jinxing Yu, Muxing Hua, Xi Chen, Zhou Zhou

**Affiliations:** 1 Diagnostic Laboratory Service, State Key Laboratory of Cardiovascular Disease, Fuwai Hospital, National Center for Cardiovascular Diseases, Chinese Academy of Medical Sciences, Peking Union Medical College, No.167, Beilishi Road, Xicheng District, Beijing, 100037 China; 2 Department of Laboratory Medicine, Yunnan Fuwai Cardiovascular Hospital, Kunming, 650000 China

**Keywords:** Dual antiplatelet therapy, Platelet function tests, High on-treatment platelet reactivity, Mean platelet volume

## Abstract

**Background:**

Knowledge on the pharmacodynamic effects of antiplatelet drugs including clopidogrel and ticagrelor on Asian patients is scarce. We aim to evaluate the effects of the two drugs on platelet reactivity in the treatment of Chinese patients who underwent percutaneous coronary intervention (PCI), using two platelet function tests (PFT). Meanwhile, the relationship between mean platelet volume (MPV), a routine index of platelet size, and high on-treatment platelet reactivity (HPR) is also investigated.

**Methods:**

Patients receiving dual antiplatelet therapy (DAPT) were scheduled for the assessment of platelet reactivity at 2–3 days after PCI. Two PFTs, light transmission aggregometry (LTA) and vasodilator-stimulated phosphoprotein (VASP)-FCM assay, were applied in the evaluation of platelet reactivity. The MPV was measured simultaneously with EDTA plasma using a Sysmex XN 2000 automated hematology analyzer.

**Results:**

The final study population included the aspirin + clopidogrel group (*n* = 46) and the aspirin + ticagrelor group (*n* = 66). In the aspirin + ticagrelor group, the maximal light transmittance (LT) changes in response to 5 μM ADP assessed by LTA was obviously lower than that in the aspirin + clopidogrel group (*P* <  0.001). The platelet reactivity index (PRI) level in the VASP test was also markedly lower in the group given aspirin and ticagrelor (P <  0.001). There was a significant difference in HPR between the two groups. MPV showed a potent ability to predict the presence of HPR at VASP assay (AUC = 0.788, 95% CI: 0.701–0.875, *P* <  0.001) in receiver-operating characteristic curve analysis.

**Conclusions:**

Compared with clopidogrel, ticagrelor has dramatically greater antiplatelet effect, with a superiority in suppressing platelet function and a lower HPR rate. In addition, there existed a significant independent association between MPV and high prevalence of HPR in the VASP assay.

**Supplementary Information:**

The online version contains supplementary material available at 10.1186/s12959-021-00350-2.

## Introduction

It is known that dual antiplatelet therapy (DAPT) which consists of aspirin and one of the P2Y12 receptor antagonists has been used for secondary prevention of thrombotic events, particularly in acute coronary syndromes (ACS) and percutaneous coronary intervention (PCI) with stenting [1, 2]. Clopidogrel, a popular P2Y12 receptor inhibitor, is most widely used. However, the wide inter- and intra-individual variability in clopidogrel response represents a significant clinical limitation [3, 4], and high on-treatment platelet reactivity (HPR) to adenosine diphosphate (ADP) is now regarded as a well-established marker reflecting the thrombotic recurrence risk [5, 6]. In comparison with clopidogrel (a thienopyridine P2Y12R antagonist), which can bind irreversibly to the ADP receptor, ticagrelor, a novel non-thienopyridine ADP antagonist, reversibly inhibits the ADP P2Y12 receptor located on platelets, preventing platelet activation and aggregation [7]. To date, most clinical studies have demonstrated that ticagrelor can provide a more rapid effect on platelet inhibition and a more favorable pharmacodynamic profile when compared with clopidogrel [8, 9]. In addition, it has been recommended in the current guideline that new DAPT with aspirin and ticagrelor can be administered to patients with ACS after stenting (IIA) [10].

It has been suggested that ethnic differences in the response to P2Y12 inhibitors could influence the pharmacodynamic effects in different races. East Asian patients are at a higher bleeding risk under DAPT, compared with non-East Asians [11]. In Korean and Japanese patients with ACS, ticagrelor presented a higher incidence of significant bleeding, compared with clopidogrel [12, 13]. Unfortunately, few Chinese patients were included in the studies that investigated the responses to clopidogrel and ticagrelor. Therefore, exploring the effects of the two popular antiplatelet drugs on platelet function in Asian/Chinese patients is increasingly urgent and will provide more potent clinical and experimental evidence to guide the use of these antiplatelet drugs among East Asian populations.

Platelet activation by ADP is central to the development of atherothrombosis. Platelet function tests (PFT) play an important role in evaluating individual antiplatelet drug responses and the therapeutic effects of different treatments. Therefore, PFT are recommended to guide the clinical treatment of patients with high risk factors for ischemia and those undergoing PCI or who have poor drug compliance [14]. In recent years, platelet reactivity has been measured by various systems in PFT [15]. Modern laboratory techniques, including the platelet function analyzer (PFA), the VerifyNow P2Y12 assay, light-transmission aggregometry (LTA), multiple electrode platelet aggregometry (MEA), thromboelastography (TEG) and vasodilator-stimulated phosphoprotein (VASP) assay, are all applied to measure different properties associated with platelet reactivity, with different detection principles. LTA, invented by Born [16, 17] and O’Brien [18], is the oldest available method for assessment of platelet activation and was regarded as the “gold standard” [19]. The VASP assay was based on flow cytometric measurement of the VASP phosphorylation level [20]. Furthermore, mean platelet volume (MPV), which is a common index indicating platelet size, has been recommended as a marker of platelet activity [21]. However, the role of MPV in evaluating HPR rates in patients with stents is still debated, because contrasting results have been reported so far on the relationship between platelet size and aggregation [22–24].

Therefore, our present study aimed to evaluate the pharmacodynamic effects of clopidogrel and ticagrelor on Chinese patients undergoing PCI, with LTA and VASP assay used to assess platelet function. We also investigated the relationship between MPV and HPR among patients receiving DAPT.

## Methods

### Study design

For this prospective study, we consecutively enrolled 112 patients presenting to Fuwai Hospital (Beijing, China) who were scheduled to undergo PCI for the assessment of platelet function from March 2017 to June 2017. The procedure followed in this study was in accordance with standard ethical principles according to the Declaration of Helsinki. Consecutive participants were enrolled in line with the following inclusion criteria: age > 18 years; had undergone PCI with drug-eluting stenting. The exclusion criteria were as follows: a history of coronary artery bypass graft or heart transplantation; use of glycoprotein IIb/IIIa receptor inhibitors or other ADP receptor antagonists; infection; renal failure undergoing dialysis; history of drug allergy; bleeding; family history and platelet count < 100 × 10^9^ cells/L. Diagnosis and performance of PCI were based on standard practices. The participants were divided into two groups: the aspirin + clopidogrel group (*n* = 46) and the aspirin + ticagrelor group (*n* = 66) according to different clinical therapy methods. The choice of DAPT depended on patients’ clinical characteristics and evaluations of clinicians, in accordance with 2017 ESC guideline [25]. For those who had received maintenance antiplatelet therapy comprising aspirin 100 mg (BAYASPIRIN®, Bayer Healthcare Manufacturing S.r.l., Garbagnate Milanese, Italy) and clopidogrel 75 mg (PLAVIX®, Sanofi Winthrop Industrie, Carbon Blanc Cedex, France) for longer than five days, clopidogrel 300-mg loading dose (LD) was administered at least 12 h before PCI. An LD of clopidogrel 300 mg and 200 mg aspirin was administered at least 12 h before PCI for those who had not received aspirin and clopidogrel antiplatelet maintenance therapy. Then daily thereafter PCI, all patients were given a maintenance dose of clopidogrel and aspirin (75 mg and 100 mg once daily) at least 1 year. An LD of ticagrelor 180 mg and a maintenance dose (90 mg twice daily) were also administered. Additional file 1 (Fig. S1) shows the flow chart of our study design.

### Clinical data collection

Patients’ basic information including age and sex was recorded on admission. The clinical characteristic information including body mass index (BMI), left ventricular ejection fraction (LVEF), history of diseases and medication were also recorded simultaneously. Patients were scheduled for biochemistry and platelet function testing at 2–3 days after drug intake, on the day of PCI. Patients took clopidogrel and aspirin after breakfast daily and were collected blood before breakfast. The timing of last ticagrelor intake was after dinner and blood collection was at next day before breakfast. The hemoglobin (Hb), platelet count (PLT) and MPV were measured using a Sysmex XN 2000 automated hematology analyzer (Sysmex, Kobe, Japan) and appropriate reagents with EDTA plasma, within 2 h after blood draw; serum low-density lipoprotein-cholesterol (LDL-C), high-sensitivity C-reactive protein (hsCRP), alanine transaminase (ALT) and aspartate transaminase (AST) were assayed using an Olympus AU-5400 biochemistry autoanalyzer (Olympus Corporation, Mishama, Japan); the value of LVEF was determined via chest X-radiography and echocardiography.

### Platelet function measurement

#### Light transmittance aggregometry

Blood samples were collected in 3.2% sodium citrate tubes (Becton-Dickinson, San Jose, CA, USA) and tests conducted within 2 h [26]. The blood was centrifuged at 120×*g* for 10 min to acquire the supernatant as platelet-rich plasma (PRP). The platelet count of PRP was unadjusted according to recommendation of ISTH consensus [27]. Subsequently, platelet-poor plasma was obtained after another centrifugation at 1500×*g* for 15 min for the remaining blood. An AG800 automatic platelet aggregation analyzer (Techlink, Biomedical Technology Co., Ltd., Beijing, China) was applied to detect platelet aggregation at 37 °C, and 5 μM ADP was used to stimulate platelets. The maximal light transmittance (LT) changes in response to 5 μM ADP from baseline, based on the reference of platelet-poor plasma, was regarded as the result of aggregation.

#### Vasodilator-stimulated phosphoprotein assay

The VASP assay was carried out by an experienced technician within 24 h. Platelet VASP kits were used to determine VASP phosphorylation according to the manufacturer’s instructions (Diagnostica Stago, Asnières, France). Briefly, blood samples were collected in 3.2% sodium citrate tubes. Fixation was performed after incubation with ADP and/or prostaglandin E_1_ (PGE_1_) in vitro. In the process of indirect immunolabeling, each sample was incubated with 16C2 monoclonal antibody and then stained with a goat anti-mouse fluorescein isothiocyanate polyclonal antibody. Flow cytometric measurement was performed via a cytometer (Mindray Bio-Medical Electronics Co., Ltd., Shenzhen, China). EPICSXL software was used to gate and analyze the mean fluorescence intensity (MFI) of platelet events. A ratio directly associated with VASP phosphorylation state was established by determining an MFI corresponding to experimental conditions. A platelet reactivity index (PRI) was calculated according to the formula: PRI = [(MFI(PGE_1_) - MFI(PGE_1_) + ADP)/MFI(PGE_1_)] × 100. The intra- and inter-assay coefficients of variation (CV) were < 5 and < 8%, respectively.

The HPR rates in the two DAPT groups were also compared. HPR was defined as those situations in which 5 μM ADP maximum platelet aggregation was ≥46% [5], or the VASP result was > 50% PRI [28].

### Statistical analysis

SPSS version 21.0 (SPSS Inc., Chicago, Illinois) was used to analyze the data statistically. For continuous variables, the results were shown as mean ± standard deviation (SD) if in a normal distribution or as median and interquartile range (IQR, percentiles 25–75) if the distribution was skewed. Statistical significance was assessed by independent-samples *t*-test, one-way analysis of variance (ANOVA), or the Mann–Whitney U test. Chi-square or Fisher exact tests were used for categorical variables and expressed as number, n (proportions, %). Bivariate correlation analysis was assessed with Pearson’s or Spearman’s correlation analysis. To identify the independent associations between MPV and HPR adjusted for hypertension, dyslipidemia, diabetes, previous PCI, angiotensin converting enzyme inhibitor (ACEI), β-blocker, LDL-C, hsCRP, and AST, multivariate logistic regression analysis was performed. Receiver-operating characteristic (ROC) curves were assessed to evaluate the predictive value of MPV for HPR. The optimal cut-off value was determined by Youden’s index (YI) calculated as (sensitivity + specificity – 1). A 2-tailed *P*-value < 0.05 indicated statistical significance.

## Results

### Study population and baseline characteristics

In total, 112 consecutive participants were enrolled in this study. Table 1 shows the baseline characteristics of the two DAPT groups. There were no significant differences in mean age, BMI and proportion of males between the two groups. The patients receiving aspirin and clopidogrel had higher LVEF, more history of hypertension and less history of diabetes. The patients treated with aspirin and ticagrelor had more previous PCI and myocardial infarction (MI) and more frequent use of ACEI. In the laboratory data acquired after PCI, the levels of hsCRP, ALT and AST were markedly higher in the group given aspirin and ticagrelor. Interestingly, patients in the aspirin + clopidogrel group had a significantly higher value of MPV (11.4 ± 1.4 vs 10.2 ± 0.9 fL, *P* <  0.001). Nevertheless, no significant difference was found in PLT count between the two groups.

**Table 1:** Demographics of the study population

Variables	Aspirin + clopidogrel group (*n* = 46)	Aspirin + ticagrelor group (*n* = 66)	*P*
Age, yrs.	**58.1 ± 9.1**	**56.3 ± 11.6**	**0.401**
Male, n (%)	**36(78.3)**	**55(83.3)**	**0.499**
BMI, kg/m^2^	**25.9 ± 3.3**	**25.9 ± 3.2**	**0.963**
LVEF, %	**63.0(60.0, 66.0)**	**60.0(52.8, 65.0)**	**< 0.001**
Risk factors			
Hypertension, n (%)	**33(71.7)**	**31(47.0)**	**0.009**
Diabetes, n (%)	**9(19.6)**	**26(39.4)**	**0.026**
Hyperlipidemia, n (%)	**43(93.5)**	**58(89.2)**	**0.441**
Past history			
Previous PCI, n (%)	**9(19.6)**	**28(42.4)**	**0.011**
Previous MI, n (%)	**17(37.0)**	**56(84.8)**	**< 0.001**
Previous stroke, n (%)	**10(22.2)**	**4(6.1)**	**0.012**
Medications			
Statins, n (%)	**46(100.0)**	**66(100.0)**	**1.000**
ACEI, n (%)	**11(24.4)**	**29(46.0)**	**0.022**
ARB, n (%)	**8(18.2)**	**7(11.1)**	**0.300**
β-blocker, n (%)	**38(84.4)**	**60(93.8)**	**0.112**
Laboratory			
Hemoglobin, g/L	**147.7 ± 14.0**	**143.5 ± 12.4**	**0.095**
LDL-C, mmol/L	**2.0(1.7, 2.8)**	**2.2(1.5, 2.7)**	**0.708**
HsCRP, mg/L	**1.1(0.5, 3.0)**	**2.6(1.2, 7.2)**	**0.006**
AST, U/L	**22.0(19.0, 27.5)**	**27.0(20.0, 85.0)**	**0.007**
ALT, U/L	**27.0(17.0, 39.2)**	**32.5(22.8, 52.5)**	**0.022**
Platelet, 10^9^ cells/L	**223.1 ± 60.2**	**244.7 ± 69.7**	**0.091**
MPV, fL	**11.4 ± 1.4**	**10.2 ± 0.9**	**< 0.001**

Values are mean ± SD if the distribution is normal; median (interquartile range) if skewed; number, n (proportions, %) for categorical variables

*ACEI* Angiotensin converting enzyme inhibitor, *ALT* Alanine transaminase, *AST* Aspartate transaminase, *ARB* Angiotensin receptor blocker, *BMI* Body mass index, *CABG* Coronary artery bypass graft, *hsCRP* highly sensitive C-reactive protein, *LDL-C* Low-density lipoprotein cholesterol, *LVEF* Left ventricular ejection fraction, *MI* Myocardial infarction, *MPV* Mean platelet volume, *PCI* Percutaneous coronary intervention

### Platelet function testing in patients of the two DAPT groups

**Fig. 1:**
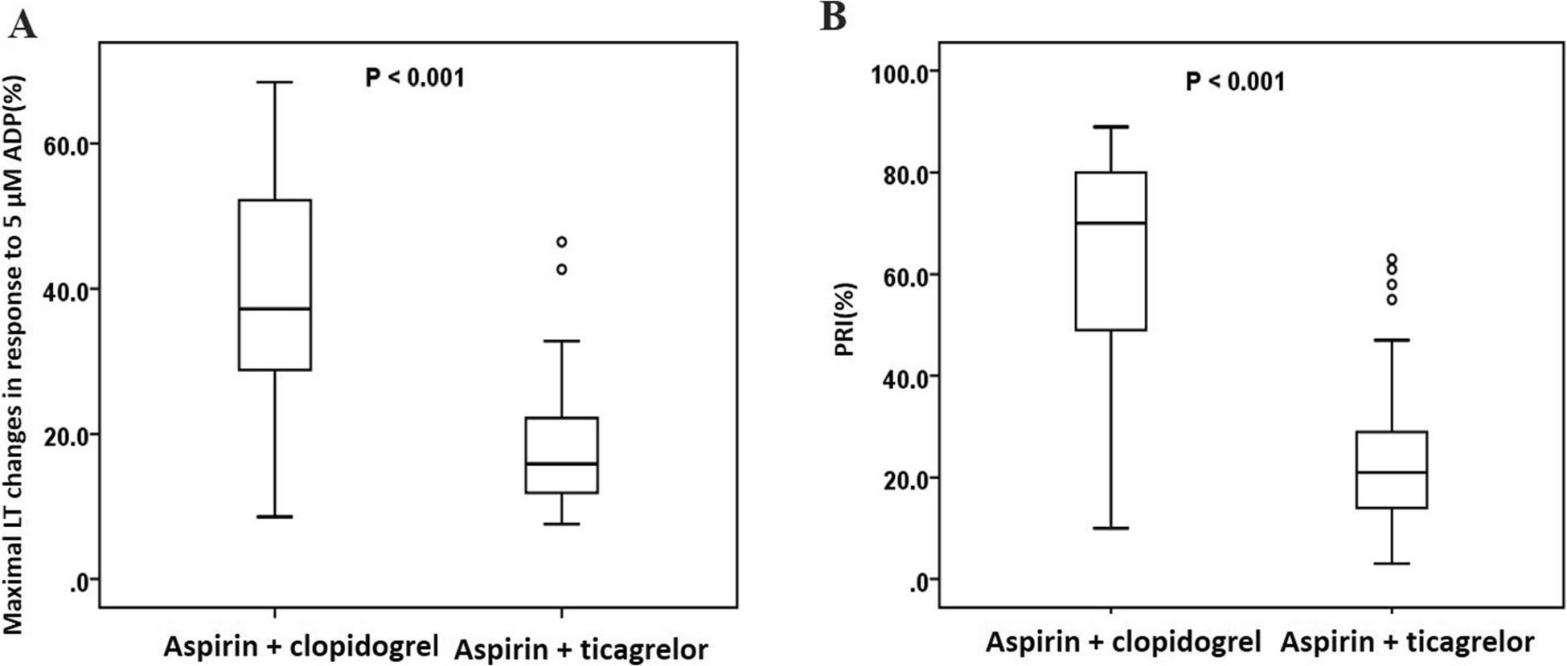
The platelet function test values for different devices, measured post-percutaneous coronary intervention (PCI). **A** Light transmittance aggregometry (maximal LT changes in response to 5 μM ADP, %); **B** Vasodilator - stimulated phosphoprotein test (PRI, %). Error bars indicate SD. Abbreviations: ADP, adenosine diphosphate; PRI, platelet response index

As measured by LTA, the maximal light transmittance (LT) changes in response to 5 μM ADP in the aspirin + clopidogrel group was dramatically higher than that in the aspirin + ticagrelor group (38.8 ± 16.2% vs 18.5 ± 9.5%, *P* < 0.001; Fig. 1A). According to the results of the VASP test, the aspirin + ticagrelor group presented a significantly lower PRI level than the aspirin + clopidogrel group (23.9 ± 15.9% vs 63.5 ± 21.5%, *P* < 0.001; Fig. 1B).

**Fig. 2:**
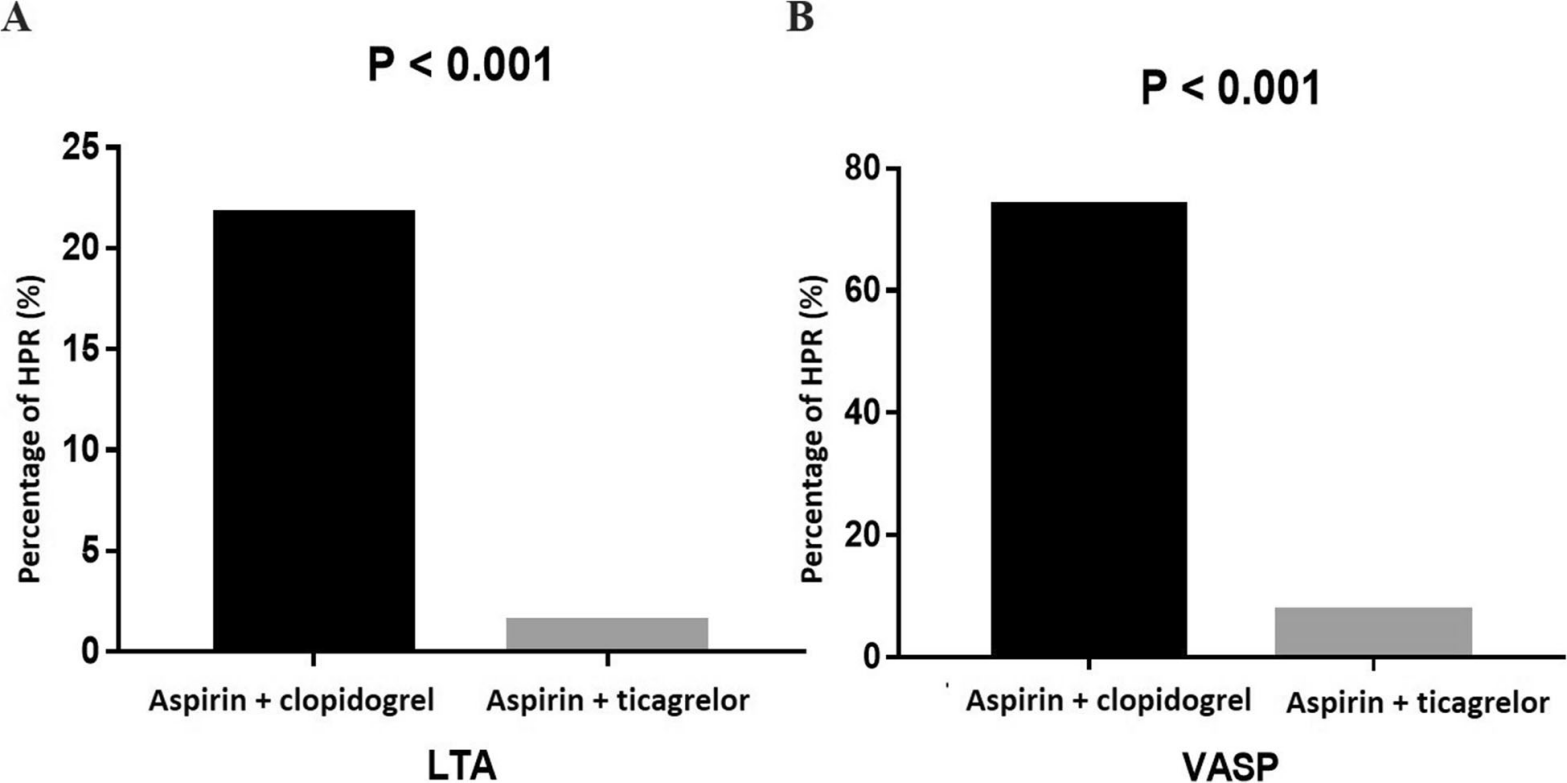
The percentage of high platelet reactivity (HPR) measured post-percutaneous coronary intervention (PCI) in different groups. **A** ADP-Light transmittance aggregometry (LTA); **B** Vasodilator-stimulated phosphoprotein (VASP) test

Using the criteria which have been reported in the literature [14, 29], the HPR rates were compared between the different DAPT groups and platelet function results (Fig. 2). In the LTA assay, the HPR rate differed significantly between the two groups (aspirin + clopidogrel: aspirin + ticagrelor = 21.7:1.5, *P* < 0.001; Fig. 2A). Similar results could also be observed in the VASP assay, with a higher HPR rate in those given aspirin and clopidogrel (aspirin + clopidogrel: aspirin + ticagrelor = 73.9:7.6, P < 0.001; Fig. 2B).

In addition, as shown in Additional file 2 (Fig. S2), a significant positive correlation was observed between the results for LTA and VASP assay (r = 0.660, *P* < 0.001). Multivariate regression analysis for prediction of HPR by the LTA and VASP assay is presented in Additional file 3 (Table S1). After multivariate adjustment for previous percutaneous coronary intervention, previous stroke, MPV, hsCRP, left ventricular ejection fraction, and aspartate transaminase, dual antiplatelet therapy remained independently significant in predicting HPR in the LTA and VASP assay.

### Association between mean platelet volume and high on-treatment platelet reactivity

**Fig. 3:**
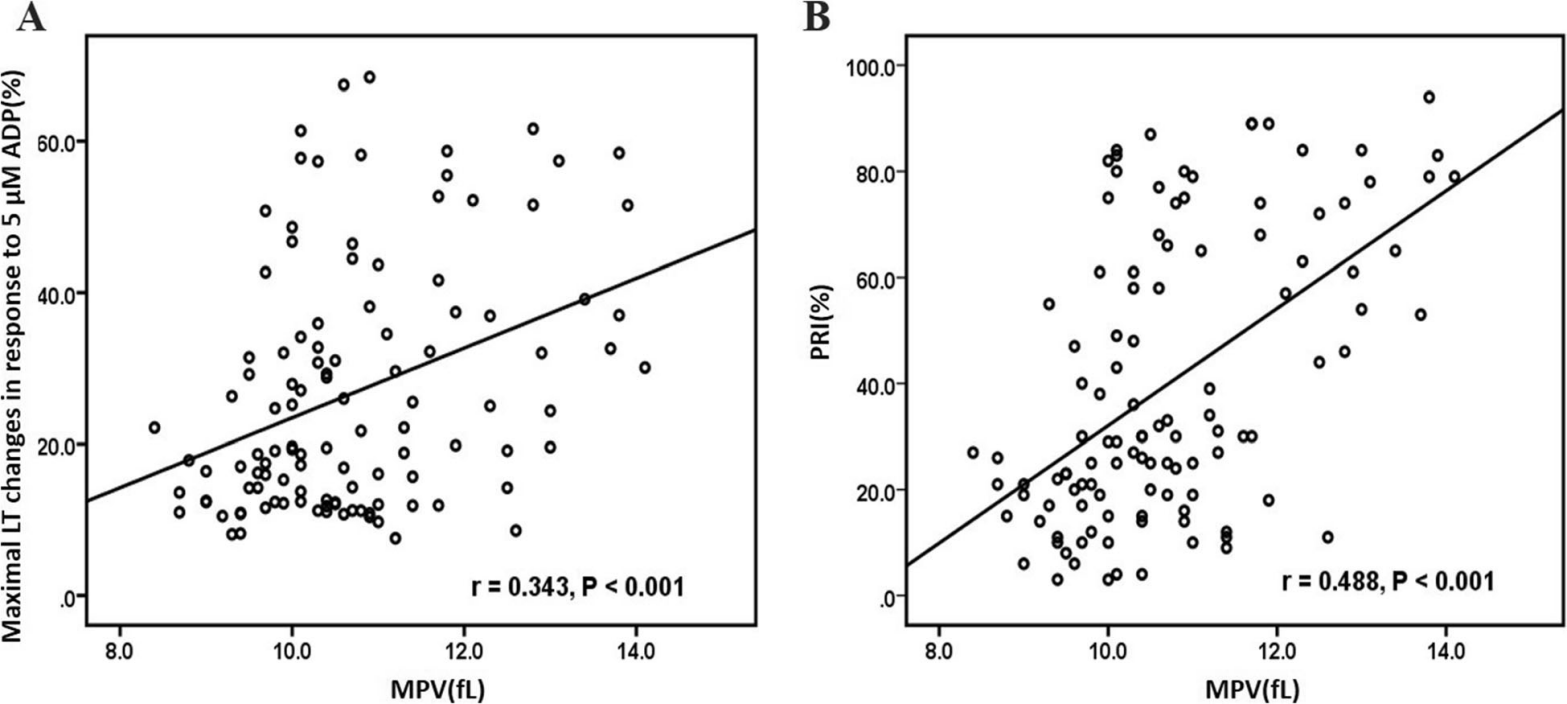
Relationships between mean platelet volume (MPV) and platelet aggregation results. **A** LTA after ADP stimulation; **B** VASP test. Correlation coefficient (r) was calculated using Pearson’s method

To investigate whether MPV could indicate the level of HPR in patients receiving DAPT, the participants were divided into three groups, according to MPV tertiles (< 10.0 fL; 10.0–11.0 fL; ≥ 11.0 fL). Table 2 shows the main clinical characteristics and biochemistry results according to the MPV values. Larger platelets were associated with higher LVEF (*P* = 0.026), higher Hb (*P* = 0.011), lower AST levels (*P* = 0.010) and lower PLT count (*P* = 0.001), and with less history of previous MI (*P* = 0.031). Notably, more patients in the highest MPV tertile received aspirin and clopidogrel (*P* < 0.001). With regard to the platelet function results, ADP-mediated platelet aggregation (*P* = 0.006) and PRI (*P* < 0.001) was much higher in patients of the highest MPV tertile. A higher percentage of HPR was observed at VASP assay in patients with higher MPV (*P* < 0.001), and this was also true for ADP-induced aggregation at LTA test (*P* = 0.048). Furthermore, analysis showed that there existed a positive correlation between MPV levels and PRI (r = 0.488, *P* < 0.001), as well as maximal LT changes in response to 5 μM ADP (r = 0.343, *P* < 0.001) (Fig. 3). There was also an inverse relationship between MPV and platelet count (r = − 0.413, *P* < 0.001).

**Table 2:** Clinical characteristics and chemistry results according to mean platelet volume

Variables	I tert < 10.0 fL (*n* = 37)	II tert 10.0–11.0 fL (*n* = 42)	III tert ≥11.0 fL (*n* = 33)	*P*
Age, yrs.	**58.2 ± 10.3**	**55.7 ± 12.1**	**57.4 ± 9.2**	**0.566**
Male, n (%)	**33(89.2)**	**31(73.8)**	**27(81.8)**	**0.216**
BMI, kg/m^2^	**26.1 ± 3.5**	**25.9 ± 3.4**	**25.5 ± 2.6**	**0.758**
LVEF, %	**60.0(53.0, 65.0)**	**63.0(60.0, 65.0)**	**65.0(58.0, 68.0)**	**0.026**
Risk factors				
Hypertension, n (%)	**20(54.1)**	**20(47.6)**	**24(72.7)**	**0.083**
Diabetes, n (%)	**14(37.8)**	**14(33.3)**	**7(21.2)**	**0.304**
Hyperlipidemia, n (%)	**34(91.9)**	**38 (90.5)**	**29(87.9)**	**0.850**
Past history				
Previous PCI, n (%)	**15(40.5)**	**12(28.6)**	**10(30.3)**	**0.489**
Previous MI, n (%)	**29(78.4)**	**28(66.7)**	**16(48.5)**	**0.031**
Previous stroke, n (%)	**4(10.8)**	**4(9.5)**	**6(18.2)**	**0.494**
Medications				
Statins, n (%)	**37(100.0)**	**42(100.0)**	**33(100.0)**	**1.000**
ACEI, n (%)	**17(45.9)**	**14(33.3)**	**9(27.3)**	**0.245**
ARB, n (%)	**6(16.2)**	**3(7.1)**	**6(18.2)**	**0.313**
β-blocker, n (%)	**36(97.3)**	**34(81.0)**	**28(84.8)**	**0.078**
Laboratory				
Hemoglobin, g/L	**140.0 ± 13.0**	**147.0 ± 13.2**	**148.8 ± 11.9**	**0.011**
LDL-C, mmol/L	**2.4(1.7, 2.8)**	**2.2(1.6, 2.8)**	**2.0(1.7, 2.2)**	**0.457**
HsCRP, mg/L	**3.1(1.2, 8.4)**	**1.8(0.9, 4.7)**	**1.5(0.5, 6.7)**	**0.232**
AST, U/L	**25.0(20.5, 85.0)**	**25.5(20.8, 38.2)**	**21.0(16.5, 28.0)**	**0.010**
ALT, U/L	**26.0(22.0, 42.5)**	**32.0(21.5, 52.0)**	**29.0(17.0, 40.0)**	**0.474**
Platelet, 10^9^ cells/L	**257.3 ± 73.4**	**243.9 ± 63.2**	**201.4 ± 48.3**	**0.001**
Maximal LT changes in response to 5 μM ADP, %	**17.0(12.4, 25.7)**	**20.6(12.1, 36.5)**	**32.0(16.4, 51.5)**	**0.006**
PRI, %	**21(11.5, 28.0)**	**31.0(23.0, 66.5)**	**61.0(30.5, 79.0)**	**< 0.001**
Percentage of HPR-LTA, %	**0(0)**	**6(14.3)**	**5(15.2)**	**0.049**
Percentage of HPR-VASP, %	**4(10.8)**	**15(35.7)**	**20(60.6)**	**< 0.001**
Dual antiplatelet therapy				
Traditional therapy, n (%)	**6(16.2)**	**18(42.9)**	**22(66.7)**	**< 0.001**
New therapy, n (%)	**31(83.8)**	**24(57.1)**	**11(33.3)**	**< 0.001**

Values are mean ± SD if the distribution is normal; median (interquartile range) if skewed; number, n (proportions, %) for categorical variables

*ADP* Adenosine diphosphate, *HPR* High on-treatment platelet reactivity, *LT* Light transmittance, *PRI* Platelet response index. Others were as Table 1

**Fig. 4:**
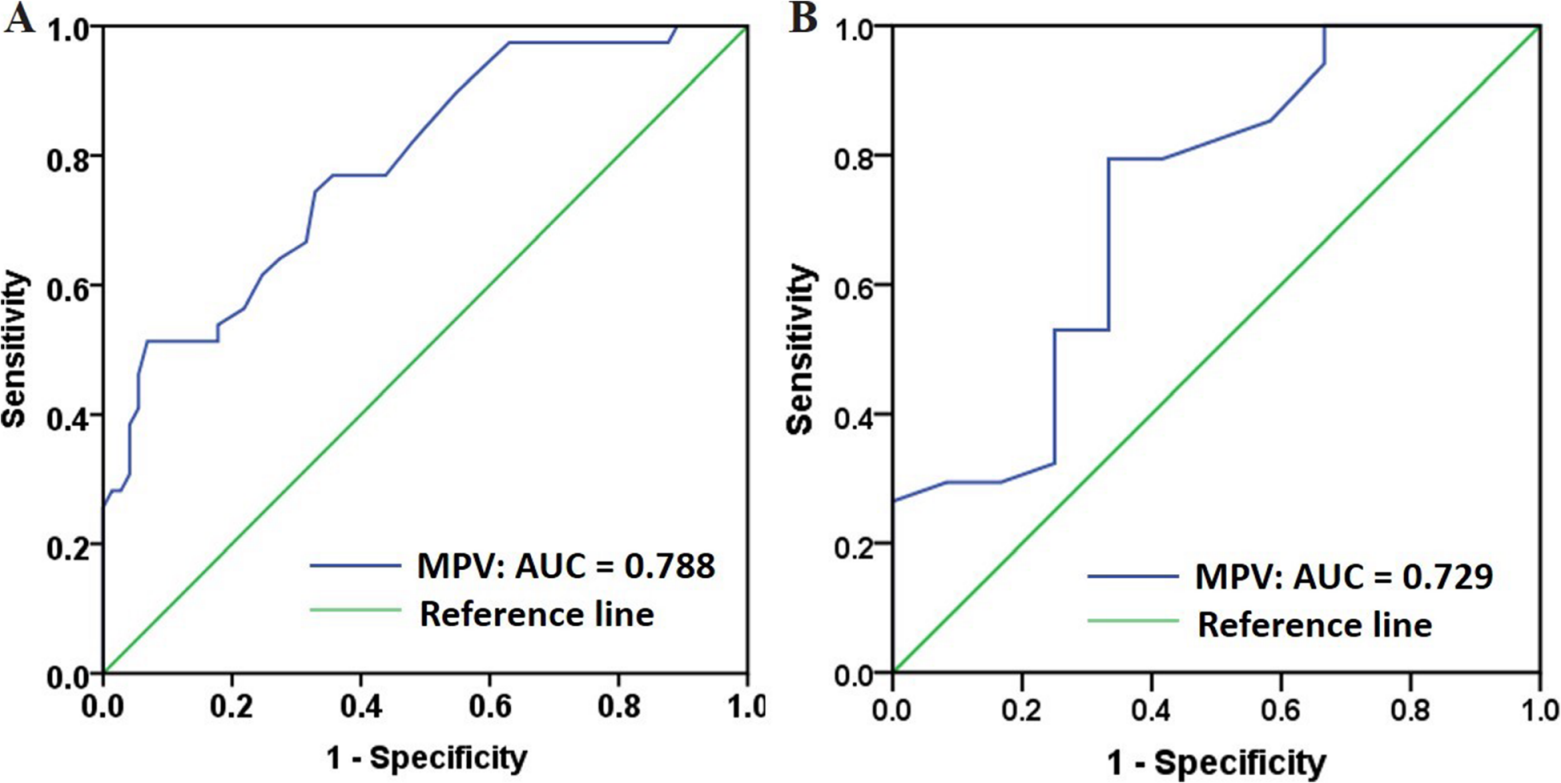
Receiver operating characteristic (ROC) curve of MPV for predicting HPR at VASP assay in different groups. **A** The whole study population. **B** Patients given aspirin and clopidogrel

As can be seen in Table 3, the independence of MPV and MPV tertiles in predicting HPR at VASP assay was subsequently determined using univariate and multivariable logistic regression analysis. Results indicated that there was an independent association between MPV level and the increased prevalence of HPR in the VASP assay, as well as MPV tertiles. As the increasing trend of MPV tertiles, it also presented higher OR values which indicating a higher prevalence of HPR. After adjustment for percutaneous coronary intervention, previous stroke, dual antiplatelet therapy, left ventricular ejection fraction and aspartate transaminase, MPV was significantly and independently associated with HPR (OR = 2.105, 95% CI:1.175–3.771, *P* = 0.012), and the results of MPV tertile 3 were marginally significant (OR = 5.446, 95% CI:0.936–31.690, *P* = 0.059). Nevertheless, MPV and MPV tertiles did not present significant independence in predicting HPR at LTA testing. In addition, analysis of ROC curves showed a potent ability of MPV in predicting the presence of HPR at VASP assay in patients undergoing PCI and receiving DAPT (area under curve [AUC] = 0.788, 95% CI: 0.701–0.875, *P* < 0.001) (Fig. 4A). The cut-off value of MPV in predicting HPR was 10.55 fL. Furthermore, analysis of ROC curves was also conducted in patients given aspirin and clopidogrel (Fig. 4B). MPV could predict HPR at VASP assay for those patients (area under curve [AUC] = 0.729, 95% CI: 0.552–0.906, *P* = 0.019) with a cut-off value of 11.65 fL.

**Table 3:** Regression analysis to assess HPR according to MPV and MPV tertiles^a^

Variables	Univariate		Multivariate	
	OR (95% CI)	P	OR (95% CI)	P
MPV	**2.797(1.813, 4.313)**	**< 0.001**	**2.105(1.175, 3.771)**	**0.012**
MPV tertiles tertiles				
Tertile 1	**1**		**1**	
Tertile 2	**4.583(1.361, 15.440)**	**0.014**	**3.537(0.644, 19.442)**	**0.146**
Tertile 3	**12.692(3.634, 44.333)**	**< 0.001**	**5.446(0.936, 31.690)**	**0.059**

^a^ Adjusted for previous of percutaneous coronary intervention, previous of stroke, dual antiplatelet therapy, left ventricular ejection fraction, and aspartate transaminase

*HPR* High on-treatment platelet reactivity, *MPV* Mean platelet volume

**Fig. 5:**
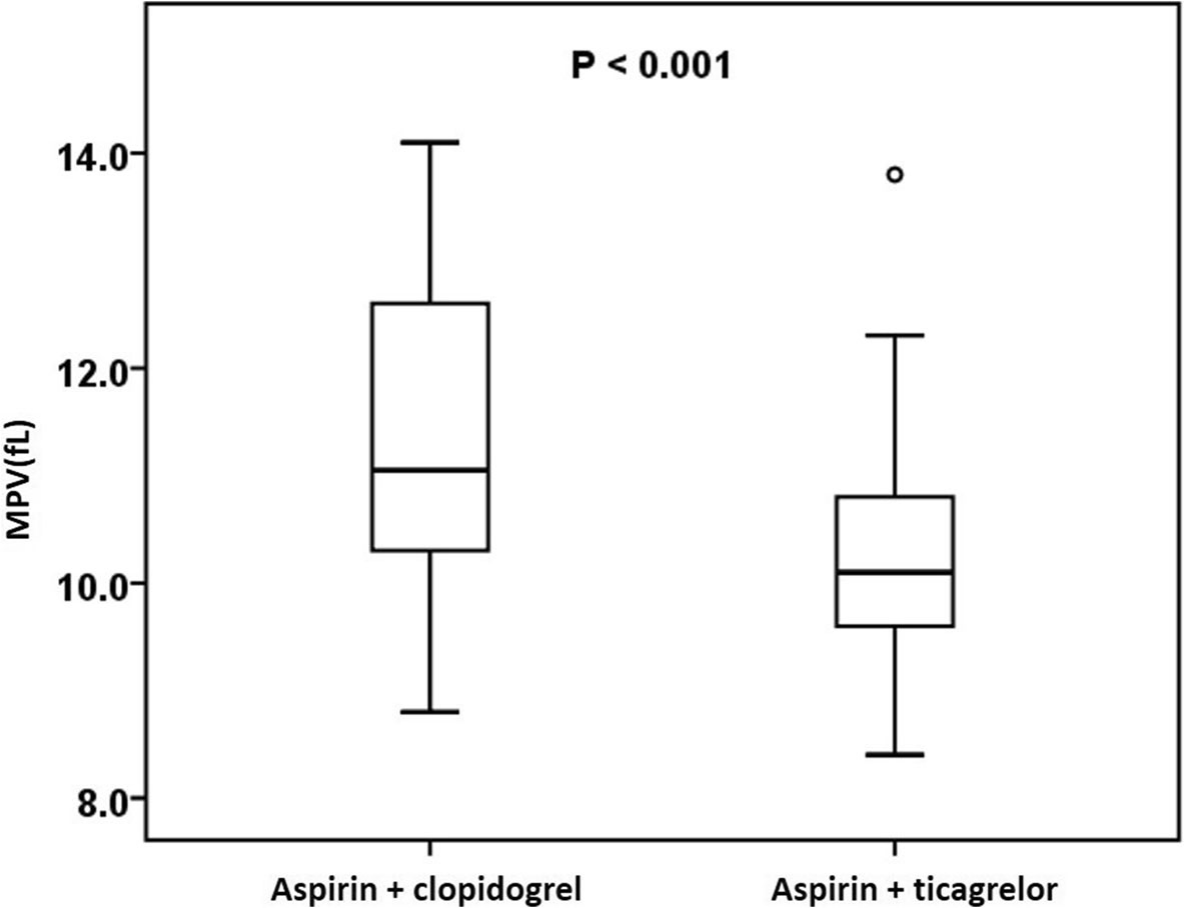
The comparison of MPV levels in the groups given aspirin and clopidogrel or ticagrelor

Finally, Fig. 5 displays the MPV levels of the two groups given aspirin and clopidogrel or ticagrelor. It was found that the levels of MPV were significantly higher in those patients given aspirin and clopidogrel than those given aspirin and ticagrelor (*P* < 0.001), which was also shown in Table 1.

## Discussion

Our study, for the first time, characterized the effects of clopidogrel and ticagrelor on platelet function, using LTA and VASP assay, in a Chinese population undergoing PCI. This study provides evidence that MPV is independently associated with HPR at VASP assay and could also be used to evaluate the platelet reactivity of patients receiving clopidogrel or ticagrelor. The three major findings in this study are as follows. (1) The effect of ticagrelor on platelet reactivity was significantly greater than that of clopidogrel, with a more potent inhibition of platelet activity measured by LTA and VASP assay. (2) Patients with ACS undergoing PCI who were received standard-of-care treatment with ticagrelor, on a basis of aspirin, had a lower prevalence of HPR when compared with those given clopidogrel. (3) MPV can independently indicate HPR in patients, measured by VASP assay, and was much higher in patients using clopidogrel, which potentially reflects a higher prevalence of HPR.

Many studies have previously assessed the pharmacodynamic effects of clopidogrel and ticagrelor in patients with ST-segment elevation myocardial infarction (STEMI) undergoing early PCI using different PFTs, and have reported that ticagrelor provided more potent and prompt platelet inhibition than clopidogrel [30]. In particular, several clinical trials demonstrated that the primary efficacy end point and clinical benefits favored ticagrelor compared with clopidogrel in patients with ACS; the former markedly reduced the mortality due to stroke, vascular causes, and myocardial infarction [31, 32]. In this study, the effect on platelet function of ticagrelor and clopidogrel in Chinese patients agreed with the findings reported in Western populations. Although there have been several studies investigating the pharmacology and bleeding risk associated with two anti-platelet drugs among Asian populations [33–36], few studies have concentrated on their different effects on platelet function in such populations, using different PFTs including LTA and VASP test, even though only Verify Now was applied to evaluate the platelet inhibition with ticagrelor versus clopidogrel in diabetic patients after PCI in the study of Zhenyu Liu et al. [37].

Various kinds of PFT have been applied to monitor platelet activity in the setting of DAPT (aspirin and clopidogrel or ticagrelor) in large clinical trials. Verify Now, LTA, MEA and flow cytometry are most intensively used among those techniques. LTA, as a traditional technique, has always been acknowledged as the most classical method. VASP phosphorylation measures activation-dependent platelet signaling. This assay requires small sample volumes and whole blood, maintaining high stability, and is dependent on the P2Y12 receptor, the site of action for clopidogrel and ticagrelor [38]. Therefore, the VASP assay has been used in many clinical trials on the background of the above characteristics. Meanwhile, it has shown a relatively good correlation with LTA results, which was also seen in our results in Fig. S2. In our study, we used LTA and VASP assay and found evidence for a significant effect of ticagrelor on platelet inhibition. In addition, use of LTA and VASP testing may identify patients who are at high risk of thrombotic events such as cardiac death and stent thrombosis during follow up [39]. However, the difference in HPR prevalence with the two tests was relatively large. According to studies reported, the results of platelet aggregation and HPR remained difference with various PFTs. It was considered that the definition standard of HPR influenced our results, especially HPR in the VASP assay which was referenced with the definition of France but not Asian population. Therefore, the definition of HPR at VASP assay might be adjusted with the data of Asians in further investigations. The association between the HPR with the two tests and the ending of patients should be followed up and the study population need to be enlarged as recommended in consensus [40, 41].

Furthermore, a higher HPR rate in patients receiving clopidogrel was observed in our study. This finding indicated that clopidogrel was more prone to induce drug resistance in Chinese individuals. This could guide a more efficient tailored therapy for those patients who were identified as at very high risk. On the genetic level, CYP2C19 polymorphism have been identified as the most prominent effector on platelet activity after clopidogrel treatment [42]. According to literature reported [43, 44], as compared with Africans and whites, East Asian population has a high prevalence of the CYP2C19 loss-of-function (LOF) genotype with CYP2C19*3 variant which was in accordance with previous results found in our laboratory center with Chinese population [45]. Additionally, the prevalence of the LOF mutations among different Asian populations also presented difference, and ticagrelor could become a substitute for clopidogrel in those with LOF mutations [46].Therefore, it could be implied that our results in Chinese population may provide additional information to Asian population. Of note, patients in this study were not assigned by randomization to clopidogrel or ticagrelor because the choice of anti-platelet drugs should obey the clinical guidelines and should be evaluated by clinicians according to patients’ symptoms. The data in Table S1 demonstrate that the results of HPR might not be influenced by differences in hsCRP, transaminases, MPV and other variables between the two groups.

Further, we provided evidence that MPV, which has been proposed as a cheap and easy-to-obtain marker of platelet size, could indicate platelet reactivity and the level of HPR in patients receiving DAPT. The inverse relationship between MPV and platelet count in our results proved the reliability of this study, which was in accordance with reported findings. A close relationship has been demonstrated between MPV and cardiovascular risk factors including obesity, diabetes mellitus, hypertension, hypercholesterolemia and other factors [47, 48]. However, data relating MPV with acute coronary and cerebrovascular events are still contrasting. Lippi et al. [49] demonstrated that there was a significant increase of MPV levels in ACS patients when compared with non-ACS patients. In addition, platelet size could predict impaired angiographic reperfusion and the death rate in patients with STEMI undergoing PCI [50]. In contrast, Tavil et al. demonstrated that MPV was related to central obesity, hypertension and hypercholesterolemia, but not to coronary artery disease (CAD), in patients referred for coronary angiography [51]. The role of MPV in indicating the response to antiplatelet drugs has also raised great debate. Asher et al. [52] documented a higher rate of HPR with larger platelets after clopidogrel loading dose received by patients with acute myocardial infarction. Kubica et al. [53] reported similar findings in patients undergoing PCI. In addition, larger-sized platelets could independently predict the risk of high residual platelet reactivity for treatment with aspirin + clopidogrel, also in patients treated with PCI [54]. However, Monica et al. [24] found no impact of larger platelet volume on the majority of platelet function tests, and in particular on ADP-mediated aggregation or the response to clopidogrel or ticagrelor. Our present study evaluated the association between MPV and platelet function in patients with DAPT and confirmed the well-established strict association of MPV with other platelet function parameters, including ADP-induced aggregation conducted by LTA and PRI in the VASP assay. Regarding the variation in the role of MPV in indicating platelet function, it has been considered that this may be related to the various PFTs used in different studies. We found that MPV was related to HPR only at the VASP test but not at the LTA assay. Similar results for the association between MPV and HPR could be found at MEA test, according to Kim et al. [54]. Of note, there was a very low correlation between the results of the MEA test and platelet volume indices, although there was a strong correlation between VASP test parameters and MPV. It could be noted that there was a significant difference in the baseline value of MPV in the two treatment arms. To avoid drawing questionable conclusions on MPV, the dual antiplatelet therapy was included in the multivariate regression analysis to assess the independence of MPV and MPV tertiles in predicting HPR. Therefore, the results obtained are rational and the conclusion we drew is also reasonable. In this study, the present results of multivariable logistic regression analysis showed that MPV as a continuous variable could independently indicate HPR at VASP assay and the results of MPV tertile 3 were marginally significant. This might because the sample size of the study is not large enough to ensure enough number of samples in MPV tertiles. It could be inferred that MPV tertiles would be significantly and independently associated with HPR if the sample size were enlarged. Therefore, the sample size should be enlarged to verify the results and investigate the role of MPV tertiles in indicating HPR in the further study. Based on the results of ROC analysis, a threshold could be found (MPV = 11.65 fL) for which a switch from clopidogrel to ticagrelor should be considered. This provided additional information and evidence regarding to the role of MPV in guiding clinical practice and anti-platelet drugs using. Of note, the swelling effect of EDTA on blood cells especially on platelet volume over the first two hours should be addressed. A significant increase in MPV could be observed in blood samples anticoagulated with EDTA over time [55]. In our study, it was recommended that MPV test should be conducted within 2 h after blood draw. Therefore, time elapsed between blood collection and testing is quite important for the accuracy of testing results which should be paid more attention [55].

It has been demonstrated that ticagrelor had superiority over clopidogrel in suppressing platelet function in patients with ACS, with a more pronounced antiplatelet effect during the initial treatment phase and during maintenance therapy [56]. Our present study is consistent with previous research. Recently, the EROSION study [57] reported that DAPT with aspirin and ticagrelor without stenting may be an option for patients with ACS caused by plaque erosion. Therefore, the potential clinical application of ticagrelor in anti-thrombotic therapy for ACS patients should not be ignored.

In this study, several limitations of the design should be stated. First, the absence of long-term follow-up of our patients should be considered, and, therefore, we cannot evaluate the impact of the two different types of DAPT on clinical outcome. Furthermore, we need to enlarge the dataset in order to improve the representativeness and reliability of the results. In addition, the impact of genetic factors, such as CYP2C19 polymorphism, on anti-platelet drug responsiveness was not evaluated in this study; the response to clopidogrel is closely associated with the polymorphism of CYP2C19. The results of this study may therefore have been confounded by the prevalence of CYP2C19 polymorphism.

## Conclusions

Our study investigated the antiplatelet effect of clopidogrel and ticagrelor in Chinese patients undergoing PCI, using two platelet function tests, LTA and VASP assay. Ticagrelor has markedly greater antiplatelet effect than clopidogrel, with a superiority in inhibiting platelet activity and a lower HPR rate. In addition, an independent association between MPV and high HPR prevalence in the VASP assay was found. Clinicians should be aware that MPV could be another potential marker to reflect the platelet reactivity in response to anti-platelet drugs and take it into consideration during antiplatelet therapy.

## Supplementary Information

**Additional file 1: Fig. S1.** Study flow diagram.

**Additional file 2: Fig. S2.** Relationships between the results obtained by light transmittance aggregometry (LTA, %) and vasodilator-stimulated phosphoprotein (VASP) (PRI, %) assay systems post-percutaneous coronary intervention (PCI). Correlation coefficient (r) was calculated using Pearson’s method.

**Additional file 3: Table S1.** Multivariate regression analysis for prediction of HPR at LTA and VASP assay.

## Data Availability

The datasets generated and/or analysed during the current study are not publicly available due to subsequent researches based on this data base not being published but are available from the corresponding author on reasonable request.

## Abbreviations

ACEI: Angiotensin converting enzyme inhibitor
ACS: Acute coronary syndromes
ADP: Adenosine diphosphate
ALT: Alanine transaminase
ANOVA: One-way analysis of variance
AST: Aspartate transaminase
BMI: Body mass index
CAD: Coronary artery disease
CV: Coefficient of variation
DAPT: Dual antiplatelet therapy
Hb: Hemoglobin
HPR: High on-treatment platelet reactivity
hsCRP: High-sensitivity C-reactive protein
IQR: Interquartile range
LDL-C: Low-density lipoprotein-cholesterol
LTA: Light-transmission aggregometry
LVEF: Left ventricular ejection fraction
MEA: Multiple electrode platelet aggregometry
MFI: Mean fluorescence intensity
MI: Myocardial infarction
MPV: Mean platelet volume
PCI: Percutaneous coronary intervention
PFA: Platelet function analyzer
PFT: Platelet function test
PGE_1_: Prostaglandin E_1_
PLT: Platelet count
PRI: Platelet reactivity index
ROC: Receiver-operating characteristic
SD: Standard deviation
STEMI: ST-segment elevation myocardial infarction
TEG: Thromboelastography
VASP: Vasodilator-stimulated phosphoprotein
YI: Youden’s index

## References

[1] Steinhubl SR, Berger PB, Mann JT, Fry ET, DeLago A, Wilmer C (2002). Early and sustained dual oral antiplatelet therapy following percutaneous coronary intervention: a randomized controlled trial. Jama..

[2] Yusuf S, Zhao F, Mehta SR, Chrolavicius S, Tognoni G, Fox KK (2001). Effects of clopidogrel in addition to aspirin in patients with acute coronary syndromes without ST-segment elevation. N Engl J Med.

[3] Gurbel PA, Bliden KP, Hiatt BL, O'Connor CM (2003). Clopidogrel for coronary stenting: response variability, drug resistance, and the effect of pretreatment platelet reactivity. Circulation..

[4] van der Heijden DJ, Westendorp IC, Riezebos RK, Kiemeneij F, Slagboom T, van der Wieken LR (2004). Lack of efficacy of clopidogrel pre-treatment in the prevention of myocardial damage after elective stent implantation. J Am Coll Cardiol.

[5] Bonello L, Tantry US, Marcucci R, Blindt R, Angiolillo DJ, Becker R (2010). Consensus and future directions on the definition of high on-treatment platelet reactivity to adenosine diphosphate. J Am Coll Cardiol.

[6] Marcucci R, Gori AM, Paniccia R, Giusti B, Valente S, Giglioli C (2009). Cardiovascular death and nonfatal myocardial infarction in acute coronary syndrome patients receiving coronary stenting are predicted by residual platelet reactivity to ADP detected by a point-of-care assay: a 12-month follow-up. Circulation..

[7] Vang JJ, Nilsson L, Berntsson P, Wissing BM, Giordanetto F, Tomlinson W (2009). Ticagrelor binds to human P2Y(12) independently from ADP but antagonizes ADP-induced receptor signaling and platelet aggregation. J Thromb Haemost.

[8] May CH, Lincoff AM (2012). Safety profile and bleeding risk of ticagrelor compared with clopidogrel. Expert Opin Drug Saf.

[9] Franchi F, Faz GT, Rollini F, Park Y, Cho JR, Thano E (2016). Pharmacodynamic effects of switching from Prasugrel to Ticagrelor: results of the prospective, randomized SWAP-3 study. JACC Cardiovasc Interv.

[10] Levine GN, Bates ER, Bittl JA, Brindis RG, Fihn SD, Fleisher LA (2016). 2016 ACC/AHA guideline focused update on duration of dual antiplatelet therapy in patients with coronary artery disease: a report of the American College of Cardiology/American Heart Association task force on clinical practice guidelines: an update of the 2011 ACCF/AHA/SCAI guideline for percutaneous coronary intervention, 2011 ACCF/AHA guideline for coronary artery bypass graft surgery, 2012 ACC/AHA/ACP/AATS/PCNA/SCAI/STS guideline for the diagnosis and Management of Patients with Stable Ischemic Heart Disease, 2013 ACCF/AHA guideline for the management of ST-elevation myocardial infarction, 2014 AHA/ACC guideline for the Management of Patients with non-ST-elevation acute coronary syndromes, and 2014 ACC/AHA guideline on perioperative cardiovascular evaluation and Management of Patients Undergoing Noncardiac Surgery. Circulation..

[11] Kang J, Park KW, Palmerini T, Stone GW, Lee MS, Colombo A (2019). Racial differences in Ischaemia/bleeding risk trade-off during anti-platelet therapy: individual patient level landmark Meta-analysis from seven RCTs. Thromb Haemost.

[12] Park DW, Kwon O, Jang JS, Yun SC, Park H, Kang DY (2019). Clinically significant bleeding with Ticagrelor versus Clopidogrel in Korean patients with acute coronary syndromes intended for invasive management: a randomized clinical trial. Circulation..

[13] Goto S, Huang CH, Park SJ, Emanuelsson H, Kimura T (2015). Ticagrelor vs. clopidogrel in Japanese, Korean and Taiwanese patients with acute coronary syndrome -- randomized, double-blind, phase III PHILO study. Circ J.

[14] Gorog DA, Fuster V (2013). Platelet function tests in clinical cardiology: unfulfilled expectations. J Am Coll Cardiol.

[15] Kong R, Trimmings A, Hutchinson N, Gill R, Agarwal S, Davidson S (2015). Consensus recommendations for using the multiplate((R)) for platelet function monitoring before cardiac surgery. Int J Lab Hematol.

[16] Born GV, Cross MJ (1963). The aggregation of blood platelets. J Physiol.

[17] Born GV (1962). Aggregation of blood platelets by adenosine diphosphate and its reversal. Nature..

[18] O’Brien JR (1962). Platelet aggregation: part II some results from a new method of study. J Clin Pathol.

[19] Breddin HK (2005). Can platelet aggregometry be standardized?. Platelets..

[20] Schwarz UR, Geiger J, Walter U, Eigenthaler M (1999). Flow cytometry analysis of intracellular VASP phosphorylation for the assessment of activating and inhibitory signal transduction pathways in human platelets--definition and detection of ticlopidine/clopidogrel effects. Thromb Haemost.

[21] Chu SG, Becker RC, Berger PB, Bhatt DL, Eikelboom JW, Konkle B (2010). Mean platelet volume as a predictor of cardiovascular risk: a systematic review and meta-analysis. J Thromb Haemost.

[22] Jakl M, Sevcik R, Ceral J, Fatorova I, Horacek JM, Vojacek J (2014). Mean platelet volume and platelet count: overlooked markers of high on-treatment platelet reactivity and worse outcome in patients with acute coronary syndrome. Anadolu Kardiyol Derg.

[23] Beyan C, Kaptan K, Ifran A (2006). Platelet count, mean platelet volume, platelet distribution width, and plateletcrit do not correlate with optical platelet aggregation responses in healthy volunteers. J Thromb Thrombolysis.

[24] Verdoia M, Pergolini P, Rolla R, Nardin M, Barbieri L, Schaffer A (2015). Mean platelet volume and high-residual platelet reactivity in patients receiving dual antiplatelet therapy with clopidogrel or ticagrelor. Expert Opin Pharmacother.

[25] Valgimigli M, Bueno H, Byrne RA, Collet JP, Costa F, Jeppsson A (2018). 2017 ESC focused update on dual antiplatelet therapy in coronary artery disease developed in collaboration with EACTS: the task force for dual antiplatelet therapy in coronary artery disease of the European Society of Cardiology (ESC) and of the European Association for Cardio-Thoracic Surgery (EACTS). Eur Heart J.

[26] Kim IS, Jeong YH, Kang MK, Koh JS, Park Y, Hwang SJ (2010). Correlation of high post-treatment platelet reactivity assessed by light transmittance aggregometry and the VerifyNow P2Y12 assay. J Thromb Thrombolysis.

[27] Cattaneo M, Cerletti C, Harrison P, Hayward CP, Kenny D, Nugent D (2013). Recommendations for the standardization of light transmission Aggregometry: a consensus of the working party from the platelet physiology subcommittee of SSC/ISTH. J Thromb Haemost..

[28] Barragan P, Bouvier JL, Roquebert PO, Macaluso G, Commeau P, Comet B (2003). Resistance to thienopyridines: clinical detection of coronary stent thrombosis by monitoring of vasodilator-stimulated phosphoprotein phosphorylation. Catheter Cardiovasc Interv.

[29] Marcucci R, Gori AM, Paniccia R, Giusti B, Valente S, Giglioli C (2010). High on-treatment platelet reactivity by more than one agonist predicts 12-month follow-up cardiovascular death and non-fatal myocardial infarction in acute coronary syndrome patients receiving coronary stenting. Thromb Haemost.

[30] Dehghani P, Lavoie A, Lavi S, Crawford JJ, Harenberg S, Zimmermann RH (2017). Effects of ticagrelor versus clopidogrel on platelet function in fibrinolytic-treated STEMI patients undergoing early PCI. Am Heart J.

[31] Wallentin L, Becker RC, Budaj A, Cannon CP, Emanuelsson H, Held C (2009). Ticagrelor versus clopidogrel in patients with acute coronary syndromes. N Engl J Med.

[32] Held C, Asenblad N, Bassand JP, Becker RC, Cannon CP, Claeys MJ (2011). Ticagrelor versus clopidogrel in patients with acute coronary syndromes undergoing coronary artery bypass surgery: results from the PLATO (platelet inhibition and patient outcomes) trial. J Am Coll Cardiol.

[33] Kang HJ, Clare RM, Gao R, Held C, Himmelmann A, James SK (2015). Ticagrelor versus clopidogrel in Asian patients with acute coronary syndrome: a retrospective analysis from the platelet inhibition and patient outcomes (PLATO) trial. Am Heart J.

[34] Li H, Butler K, Yang L, Yang Z, Teng R (2012). Pharmacokinetics and tolerability of single and multiple doses of ticagrelor in healthy Chinese subjects: an open-label, sequential, two-cohort, single-Centre study. Clin Drug Investig..

[35] Wang Y, Wu H, Chen Y, Wang Q, Qian J, Ge J (2020). Ticagrelor pharmacokinetics and pharmacodynamics in Chinese patients with STEMI and NSTEMI without opioid administration. Adv Ther.

[36] Tang XF, Han YL, Zhang JH, Wang J, Zhang Y, Xu B (2015). Comparing of light transmittance aggregometry and modified thrombelastograph in predicting clinical outcomes in Chinese patients undergoing coronary stenting with clopidogrel. Chin Med J.

[37] Liu Z, Tian R, Wang Y, Chen Q, Li J, Xu L (2020). Platelet inhibition with Ticagrelor versus Clopidogrel in diabetic patients after percutaneous coronary intervention for chronic coronary syndromes. Thromb Haemost.

[38] Spurgeon BE, Aburima A, Oberprieler NG, Tasken K, Naseem KM (2014). Multiplexed phosphospecific flow cytometry enables large-scale signaling profiling and drug screening in blood platelets. J Thromb Haemost.

[39] Valenti R, Marcucci R, Capodanno D, De Luca G, Migliorini A, Gori AM (2015). Residual platelet reactivity to predict long-term clinical outcomes after clopidogrel loading in patients with acute coronary syndromes: comparison of different cutoff values by light transmission aggregometry from the responsiveness to clopidogrel and stent thrombosis 2-acute coronary syndrome (RECLOSE 2-ACS) study. J Thromb Thrombolysis.

[40] Tantry US, Bonello L, Aradi D, Price MJ, Jeong YH, Angiolillo DJ (2013). Consensus and update on the definition of on-treatment platelet reactivity to adenosine diphosphate associated with ischemia and bleeding. J Am Coll Cardiol.

[41] Aradi D, Kirtane A, Bonello L, Gurbel PA, Tantry US, Huber K (2015). Bleeding and stent thrombosis on P2Y12-inhibitors: collaborative analysis on the role of platelet reactivity for risk stratification after percutaneous coronary intervention. Eur Heart J.

[42] Jeong YH, Tantry US, Kim IS, Koh JS, Kwon TJ, Park Y (2011). Effect of CYP2C19*2 and *3 loss-of-function alleles on platelet reactivity and adverse clinical events in east Asian acute myocardial infarction survivors treated with clopidogrel and aspirin. Circ Cardiovasc Interv.

[43] Li S, Choi JL, Guo LZ, Goh RY, Kim BR, Woo KS (2016). Correlation between the CYP2C19 phenotype status and the results of three different platelet function tests in cardiovascular disease patients receiving antiplatelet therapy: an emphasis on newly introduced platelet function analyzer-200 P2Y test. Ann Lab Med.

[44] Man M, Farmen M, Dumaual C, Teng CH, Moser B, Irie S (2010). Genetic variation in metabolizing enzyme and transporter genes: comprehensive assessment in 3 major east Asian subpopulations with comparison to Caucasians and Africans. J Clin Pharmacol.

[45] Wang Z, Liu Z, Wang W, Fu Y, Chen W, Li W (2019). Two common mutations within CYP2C19 affected platelet aggregation in Chinese patients undergoing PCI: a one-year follow-up study. Pharmacogenomics J.

[46] Narasimhalu K, Ang YK, Tan DSY, De Silva DA, Tan KB (2020). Cost effectiveness of genotype-guided antiplatelet therapy in Asian ischemic stroke patients: Ticagrelor as an alternative to Clopidogrel in patients with CYP2C19 loss of function mutations. Clin Drug Investig.

[47] Nadar SK, Blann AD, Kamath S, Beevers DG, Lip GY (2004). Platelet indexes in relation to target organ damage in high-risk hypertensive patients: a substudy of the Anglo-Scandinavian cardiac outcomes trial (ASCOT). J Am Coll Cardiol.

[48] Ozder A, Eker HH (2014). Investigation of mean platelet volume in patients with type 2 diabetes mellitus and in subjects with impaired fasting glucose: a cost-effective tool in primary health care?. Int J Clin Exp Med.

[49] Lippi G, Meschi T, Borghi L (2012). Mean platelet volume increases with aging in a large population study. Thromb Res.

[50] Huczek Z, Kochman J, Filipiak KJ, Horszczaruk GJ, Grabowski M, Piatkowski R (2005). Mean platelet volume on admission predicts impaired reperfusion and long-term mortality in acute myocardial infarction treated with primary percutaneous coronary intervention. J Am Coll Cardiol.

[51] Tavil Y, Sen N, Yazici HU, Hizal F, Abaci A, Cengel A (2007). Mean platelet volume in patients with metabolic syndrome and its relationship with coronary artery disease. Thromb Res.

[52] Asher E, Fefer P, Shechter M, Beigel R, Varon D, Shenkman B (2014). Increased mean platelet volume is associated with non-responsiveness to clopidogrel. Thromb Haemost.

[53] Kubica A, Kasprzak M, Siller-Matula J, Kozinski M, Pio Navarese E, Obonska K (2014). Time-related changes in determinants of antiplatelet effect of clopidogrel in patients after myocardial infarction. Eur J Pharmacol.

[54] Kim YG, Suh JW, Yoon CH, Oh IY, Cho YS, Youn TJ (2014). Platelet volume indices are associated with high residual platelet reactivity after antiplatelet therapy in patients undergoing percutaneous coronary intervention. J Atheroscler Thromb.

[55] Hardy M, Lessire S, Kasikci S, Baudar J, Guldenpfennig M, Collard A (2020). Effects of time-interval since blood draw and of anticoagulation on platelet testing (count, indices and impedance aggregometry): a systematic study with blood from healthy volunteers. J Clin Med.

[56] Storey RF, Angiolillo DJ, Patil SB, Desai B, Ecob R, Husted S (2010). Inhibitory effects of ticagrelor compared with clopidogrel on platelet function in patients with acute coronary syndromes: the PLATO (PLATelet inhibition and patient outcomes) PLATELET substudy. J Am Coll Cardiol.

[57] Jia H, Dai J, Hou J, Xing L, Ma L, Liu H (2017). Effective anti-thrombotic therapy without stenting: intravascular optical coherence tomography-based management in plaque erosion (the EROSION study). Eur Heart J.

