# Supplementary material for: Clopidogrel versus ticagrelor in the treatment of Chinese patients undergoing percutaneous coronary intervention: effects on platelet function assessed by platelet function tests and mean platelet volume
Source: Thromb J. 2021 Dec 7;19:97. doi: 10.1186/s12959-021-00350-2 (PMC8650403; doi:10.1186/s12959-021-00350-2)
Supplement: Supplementary file 1 — Additional file 1: Fig. S1. Study flow diagram. [file 12959_2021_350_MOESM1_ESM.pdf]

Additional file 1

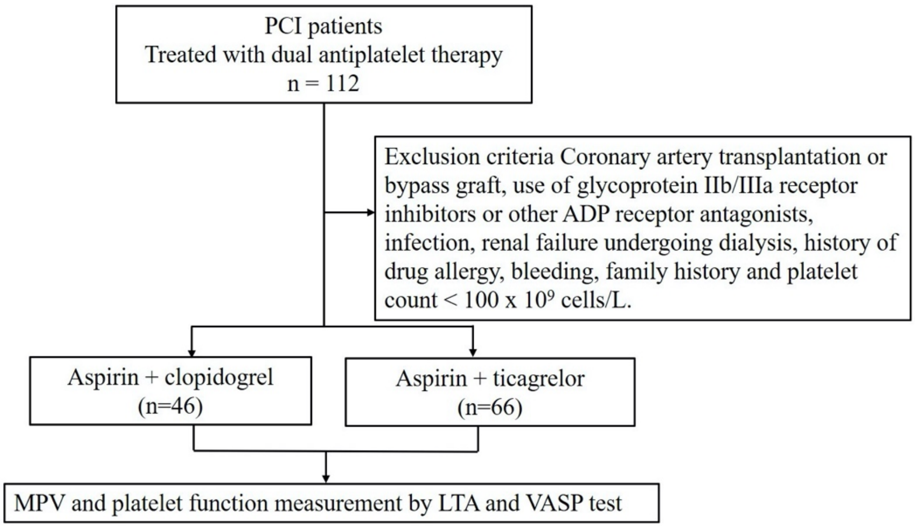

Fig. S1. Study flow diagram.

Abbreviations: PCI, percutaneous coronary intervention; MPV, mean platelet volume; LTA, light transmission aggregometry; VASP, vasodilator-stimulated phosphoprotein.
