# Supplementary material for: Clopidogrel versus ticagrelor in the treatment of Chinese patients undergoing percutaneous coronary intervention: effects on platelet function assessed by platelet function tests and mean platelet volume
Source: Thromb J. 2021 Dec 7;19:97. doi: 10.1186/s12959-021-00350-2 (PMC8650403; doi:10.1186/s12959-021-00350-2)
Supplement: Supplementary file 2 — Additional file 2: Fig. S2. Relationships between the results obtained by light transmittance aggregometry (LTA, %) and vasodilator-stimulated phosphoprotein (VASP) (PRI, %) assay systems post-percutaneous coronary intervention (PCI). Correlation coefficient (r) was calculated using Pearson’s method. [file 12959_2021_350_MOESM2_ESM.pdf]

Additional file 2

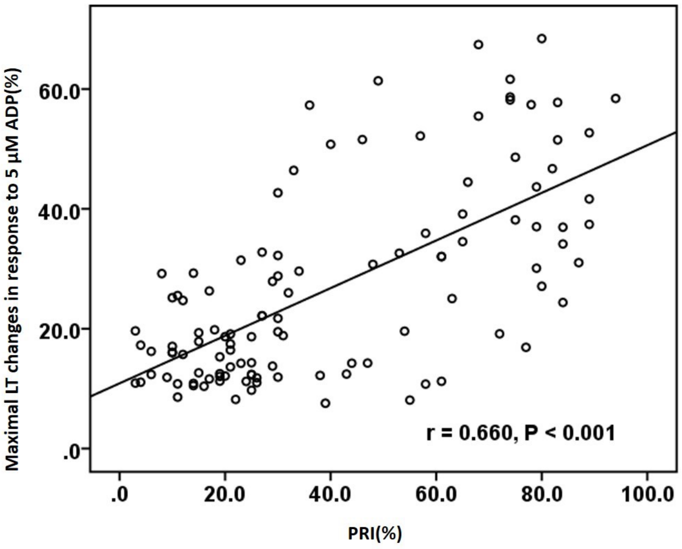

Fig. S2. Relationships between the results obtained by light transmittance aggregometry (maximal LT changes in response to 5 μM ADP, %) and vasodilator-stimulated phosphoprotein (VASP) (PRI, %) assay systems post-percutaneous coronary intervention (PCI). Correlation coefficient (r) was calculated using Pearson's method.
