# Supplementary material for: Clopidogrel versus ticagrelor in the treatment of Chinese patients undergoing percutaneous coronary intervention: effects on platelet function assessed by platelet function tests and mean platelet volume
Source: Thromb J. 2021 Dec 7;19:97. doi: 10.1186/s12959-021-00350-2 (PMC8650403; doi:10.1186/s12959-021-00350-2)
Supplement: Supplementary file 3 — Additional file 3: Table S1. Multivariate regression analysis for prediction of HPR at LTA and VASP assay. [file 12959_2021_350_MOESM3_ESM.pdf]

Table S1. Multivariate regression analysis for prediction of HPR at LTA and VASP assay.[a]

Variables	LTA		VASP assay	
	OR (95% CI)	P	OR (95% CI)	P
**dual antiplatelet therapy**	**0.048(0.004, 0.531)**	**0.013**	**0.033(0.007, 0.163)**	**<0.001**

[a] Adjusted for previous of percutaneous coronary intervention, previous of stroke, MPV, hsCRP, left ventricular ejection fraction, and aspartate transaminase.

HPR, high on-treatment platelet reactivity; MPV, mean platelet volume.
